# Robot-Assisted Removal of a Partially Intravesical Intrauterine Device (IUD) and Large Bladder Stone

**DOI:** 10.1155/2023/8074689

**Published:** 2023-01-27

**Authors:** Cassandra Heaney, Paul Lee, Andrew Winer

**Affiliations:** Department of Urology, SUNY Downstate Health Sciences University, Brooklyn, NY 11203, USA

## Abstract

An intrauterine device (IUD) is a highly effective and widely utilized option for long-acting reversible contraception. IUDs are generally well-tolerated with a low rate of serious complications. Perforation of an IUD through the uterine wall and into the urinary bladder is a rare event that may be asymptomatic. The approach for surgical removal primarily depends on the location of the device. We present a case report of a 41-year-old woman who was found to have a partially intravesical IUD and associated 2.4 cm bladder calculus. Removal of the intravesical IUD and stone was achieved with cystoscopy, cystolitholapaxy, and robot-assisted laparoscopic cystotomy.

## 1. Introduction

Intrauterine devices (IUDs) are highly effective for long-acting reversible contraception and are generally well-tolerated with a low rate of serious complications. Perforation of an IUD through the uterine wall and into the urinary bladder is rare. We describe our experience using cystoscopy, cystolitholapaxy, and robot-assisted laparoscopic cystotomy to remove a partially intravesical IUD and associated 2.4 cm bladder calculus.

## 2. Case

A 41-year-old woman with a history of recurrent urinary tract infections over the past year presented to a city hospital with one week of dysuria and three days of right flank pain radiating to the groin, associated fever, nausea, myalgias, urinary urgency, frequency, hematuria, and malodorous urine. Reproductive history was significant for seven pregnancies which resulted in five full-term births and two abortions. Her only other medical and surgical history was that of intrauterine device placement and documented removal.

**Figure 1:**
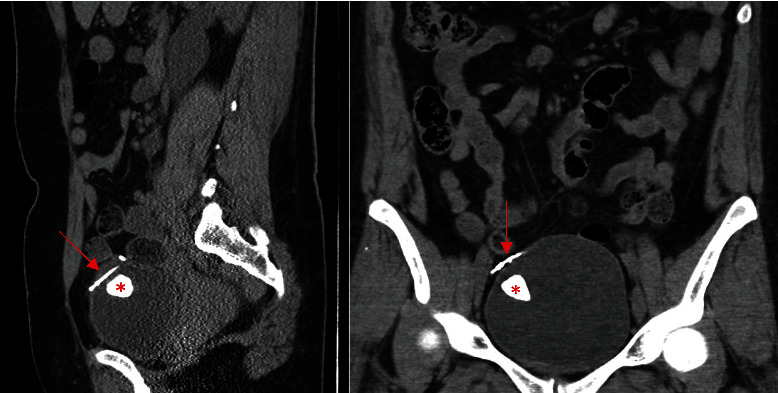
Sagittal and coronal noncontrast CT images showing large bladder calculus (red asterisk) and portion of partially intravesical T-shaped foreign body lodged in right anterior bladder wall (red arrow).

In the emergency department, she was febrile to 101.1°F and hemodynamically stable. She had lower abdominal tenderness and right costovertebral angle tenderness. Urinalysis showed moderate leukocyte esterase, 30-50 WBCs, and moderate bacteria. Urine and blood cultures grew *Enterococcus faecalis*. A noncontrast CT scan was performed and revealed a 2.4 cm bladder calculus and an approximately 3 cm T-shaped foreign body perforating the right anterior aspect of the urinary bladder (Figure 1). A likely diagnosis of IUD translocation into the bladder was made. Given the radiographic findings, surgical removal of the device and bladder repair was recommended.

**Figure 2:**
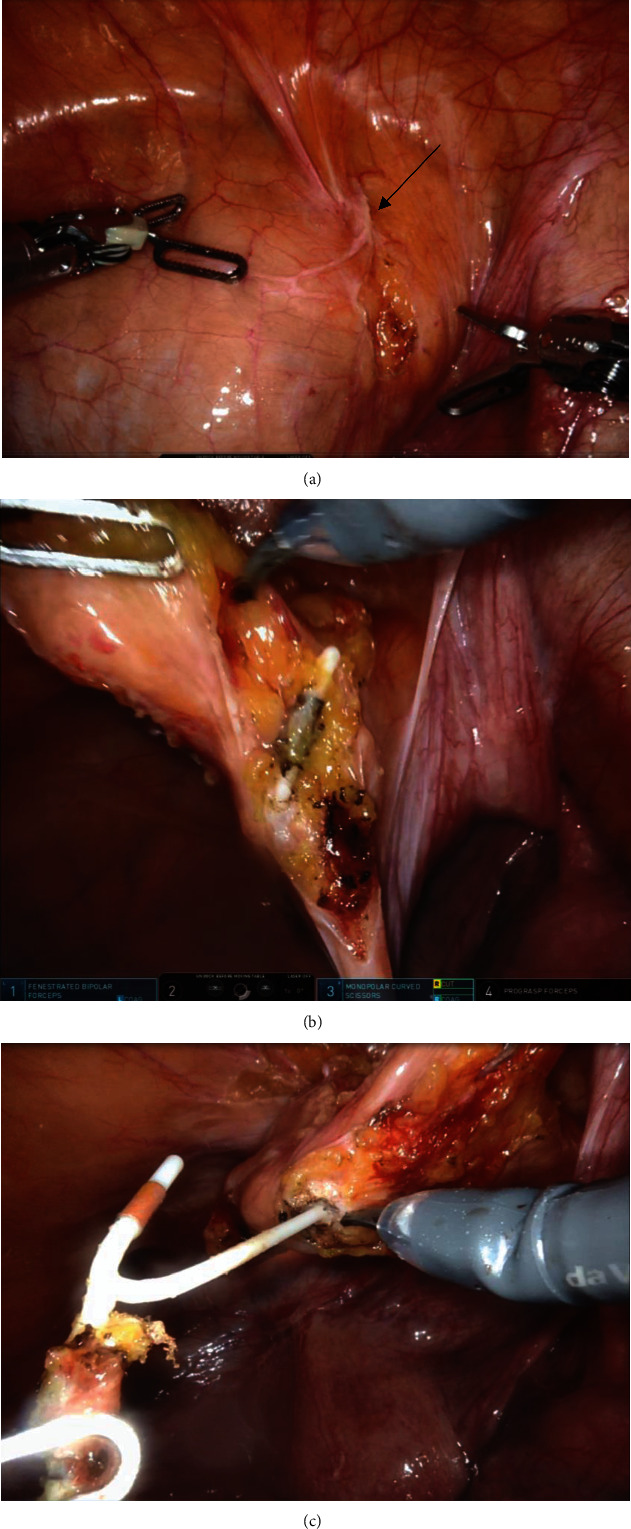
(a) Scarred peritoneum overlying the right anterior bladder dome at site of likely IUD translocation (arrow). (b) After partially entering the space of Retzius, the extravesical part of the IUD was identified. (c) A subcentimeter cystotomy was made to free the intravesical portion of the IUD and facilitate removal of the device.

**Figure 3:**
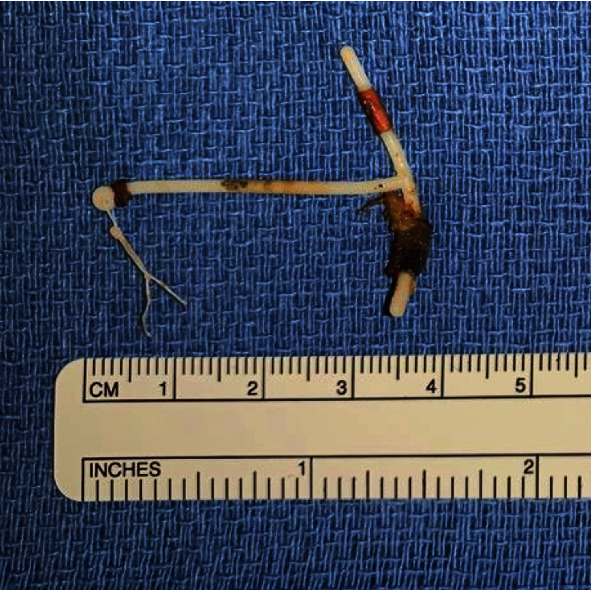
IUD specimen following surgical removal from the bladder.

The patient subsequently underwent a robot-assisted laparoscopic cystotomy, removal of bladder foreign body, cystorraphy, and transurethral cystolitholapaxy. With the patient positioned in low lithotomy to allow for transurethral access, abdominopelvic access was also obtained using the da Vinci robot. A rigid cystoscope was inserted, and the bladder inspected. The large stone was seen attached to the strings of the IUD intravesically. A Holmium:YAG laser was used to perform a cystolitholapaxy until the stone was dusted, revealing the device strings and tail emerging from the right anterior bladder wall. Gentle pull of the intravesical strings was attempted, but no movement of the device was noted. Robotically, the urinary bladder was dropped by partially entering the space of Retzius. With guidance of the cystoscope light for transillumination, the IUD was identified extravesically with robotic assistance (Figures 2(a) and 2(b)). A subcentimeter cystotomy was made and the intact IUD was removed from the bladder (Figures 2(c) and 3). Cystorraphy was performed with a figure-of-8 stitch. The patient recovered uneventfully and was discharged home the same day.

## 3. Discussion

An IUD is a safe, reliable, and cost-effective [1] option for long-acting reversible contraception. In 2019, IUDs have been estimated to be in use by over 150 million women worldwide [2]. The most common complications of IUDs are related to menstruation, such as dysmenorrhea or menorrhagia [3]. More serious complications are much less frequent, and uterine perforation is rare.

Results of large studies indicate that the rate of perforation may range from 0.4 to 1.6 per 1000 insertions. The most common mechanism of IUD perforation is iatrogenic and involves the device being driven into or through the uterine wall at the time of placement, which is more likely to occur if the procedure is complex [4, 5]. Interestingly, this complication may go unrecognized as it can often be asymptomatic [4]. Another potential mechanism involves secondary perforation caused by progressive erosion through the myometrium, which has been established in a previous report with serial CT scans over two years documenting the device's translocation [6]. This type of secondary perforation may occur due to uterine contractions [5].

It is difficult to determine when and how IUD perforation occurred in the present case. First, there is no available documentation of the perforated device's placement. In addition, she had multiple pregnancies after the removal of another IUD in 2012, and transabdominal and subsequent transvaginal pelvic ultrasounds did not document any evidence of the perforated device. This suggests that the perforated IUD may have been extrauterine for years and does not exclude or differentiate between perforation at the time of insertion or gradual translocation over time. If the patient had not required CT imaging of the abdomen and pelvis for evaluation of nephrolithiasis, it may have continued to go unrecognized.

Symptoms of a perforated IUD are generally related to the ectopic location of the device and any resulting complications. In this patient's case, the IUD had become partially embedded in the right anterior bladder wall with a portion of the tail and strings penetrating intravesically. The strings then became a nidus for the formation of a large bladder calculus. There have been numerous reports of IUDs translocating into the urinary bladder [7–14], many of which describe encrustation or stone formation around the device. This may lead to the common symptoms suggestive of bladder calculi, including irritative symptoms, hematuria, and urinary tract infections, all of which occurred in our patient's case.

The surgical approach for removing an IUD from the urinary bladder depends primarily on the location of the device. If the IUD is only partially in the bladder and strings are still visible from the cervix, it may be possible to extract the device vaginally [15]. Most published cases report cystoscopic removal, which is appropriate particularly if all or most of the IUD is intravesical. If the device is in the peritoneal cavity or partially embedded in the bladder wall, it may be extracted with laparoscopy alone or in combination with cystoscopy, as recently suggested by Liu et al. [10]. In some cases, open laparotomy may be required. The present report is the first to describe the combined use of cystoscopy, cystolitholapaxy, and robot-assisted laparoscopy to remove a partially intravesical IUD and its associated 2.4 cm calculus. This approach was beneficial for several reasons: first, cystoscopy allowed inspection of the bladder wall for the portion of the device and surrounding stone. Second, cystolitholapaxy allowed us to reduce the stone size to fragments capable of spontaneous passage while also freeing the intravesical portion of the IUD for removal through a subcentimeter cystotomy. By performing cystoscopy and robotic laparoscopy concurrently, we could precisely locate the optimal location for cystotomy and perform the incision and repair under direct vision. In cases like the present, we recommend this approach if individual patient characteristics and facility resources permit.

## Data Availability

Additional data regarding this case is not publicly available in order to protect patient privacy.
